# Structural Properties and Water Uptake of SrTi_1−x_Fe_x_O_3−x/2−δ_

**DOI:** 10.3390/ma13040965

**Published:** 2020-02-21

**Authors:** Tadeusz Miruszewski, Kacper Dzierzgowski, Piotr Winiarz, Sebastian Wachowski, Aleksandra Mielewczyk-Gryń, Maria Gazda

**Affiliations:** Department of Solid State Physics, Faculty of Applied Physics and Mathematics, Gdańsk University of Technology, Narutowicza 11/12, 80-233 Gdańsk, Poland; tadeusz.miruszewski1@pg.gda.pl (T.M.); kacper.dzierzgowski@pg.edu.pl (K.D.); sebastian.wachowski@pg.edu.pl (S.W.); alegryn@pg.edu.pl (A.M.-G.); maria.gazda@pg.edu.pl (M.G.)

**Keywords:** strontium titanate, strontium ferrite, triple conductor, protonic conductivity, water uptake

## Abstract

In this work, Fe-doped strontium titanate SrTi_1−x_Fe_x_O_3−x/2−δ_, for x = 0–1 (STFx), has been fabricated and studied. The structure and microstructure analysis showed that the Fe amount in SrTi_1−x_Fe_x_O_3−x/2−δ_ has a great influence on the lattice parameter and microstructure, including the porosity and grain size. Oxygen nonstoichiometry studies performed by thermogravimetry at different atmospheres showed that the Fe-rich compositions (x > 0.3) exhibit higher oxygen vacancies concentration of the order of magnitude 10^22^–10^23^ cm^−3^. The proton uptake investigations have been done using thermogravimetry in wet conditions, and the results showed that the compositions with x < 0.5 exhibit hydrogenation redox reactions. Proton concentration at 400 °C depends on the Fe content and was estimated to be 1.0 × 10^−2^ mol/mol for SrTi_0.9_Fe_0.1_O_2.95_ and 1.8 × 10^−5^ mol/mol for SrTi_0.5_Fe_0.5_O_2.75_. Above 20 mol% of iron content, a significant drop of proton molar concentrations at 400 °C was observed. This is related to the stronger overlapping of Fe and O orbitals after reaching the percolation level of approximately 30 mol% of the iron in SrTi_1−x_Fe_x_O_3−x/2−δ_. The relation between the proton concentration and Fe dopant content has been discussed in relation to the B-site average electronegativity, oxygen nonstoichiometry, and electronic structure.

## 1. Introduction

In recent years, mixed ionic–electronic conductors (MIECs) with relatively high electronic and ionic transfer numbers have attracted much attention. Especially perovskite-type oxides (ABO_3_) are considered as promising compounds. Their ability to stabilize higher oxidation states of B-site transition metal cations while maintaining the perovskite-type structure at high defect concentration makes them good candidates for use as electrodes in a solid-oxide fuel cells (SOFCs) [1], membranes [2], sensors [3] or other electrochemical devices [4].

Strontium titanate (SrTiO_3−δ_-STO) and strontium ferrite (SrFeO_3−δ_-SFO) have been attracting attention thanks to their unique charge transport properties and stability in different oxygen partial pressure ranges. Strontium titanate is a wide-gap semiconductor (E_g_ = 3.2 eV at T = 0 K) with low catalytic activity [5]; however, its electronic and/or ionic conductivity may be modified by aliovalent doping. Donor dopant introduced into SrTiO_3_, *i.a.* Y^3+^ [6], La^3+^ [7] or Pr^n+^ [8] on the Sr^2+^ or Nb^5+^ on theTi^4+^ [9,10] site, is compensated by the formation of cation vacancies and electrons in the conduction band [11]. On the other hand, the presence of acceptor dopants (Fe^n+^ [12,13,14], Cr^3+^ [15], Sc^3+^ [16], Al^3+^ [17], Co^n+^ [18]) leads to forming either ionized oxygen vacancies or electron holes. Therefore, a proper selection of the doping strategy enables tuning of the concentration of ionic and electronic charge carriers. Moreover, SrTiO_3_ has good chemical stability at a wide oxygen partial pressure range [19], which makes the material feasible in many applications in electrochemical devices.

Strontium ferrite (SrFeO_3−δ_) is a mixed ionic–electronic conductor that exhibits metallic-type conduction with high electrical conductivity and high oxygen ionic conductivity at oxidizing atmosphere (electronic and ionic conductivities are approximately 10^3^ S/cm and 0.2 S/cm at 850 °C in the air, respectively) [20]. Iron cations in this material are in a mixed-valence state (in a range from +3 to +4), which corresponds to the oxygen nonstoichiometry [21,22]. In contrast to SrTiO_3_, this material is unstable under low pO_2_, where the tetravalent iron cations reduce to the trivalent form (Fe^3+^), which leads to the long-range ordering of oxygen vacancies. In this case, the composition changes to SrFeO_2.5_ and the structure changes from cubic perovskite to orthorhombic brownmillerite [21,23]. In applications, it is not favorable, because the phase transition is associated with an oxygen ionic conductivity decrease as well as the mobility and concentration of holes [24].

The SrTi_1−x_Fe_x_O_3−δ_, the solid solution of SrTiO_3_ and SrFeO_3_ in the range of 0 < x < 1, was first reported in 1968 by Brixner [25] and represents a model of the mixed ionic–electronic conductor. In the SrTi_1−x_Fe_x_O_3−x/2−δ_ family of materials, oxygen ionic and electronic conductivities depend on the iron content [13,26]. Starting from the compositions with low iron content, it has been found that when titanium tetravalent cations in SrTiO_3_ are substituted by a trivalent iron, compensation occurs through oxygen vacancies in the oxygen sublattice, which influences the electronic properties of the material.

Only a few reports have been published concerning the hydration, hydrogenation, and proton conductivity of strontium titanate, strontium ferrite, and solid solutions so far. In 2002, Wideroe et al. [27] analyzed the proton conduction by the EMF (Electromotorical Force Method) and water uptake in Al-doped SrTiO_3_ by thermogravimetric measurements. They concluded that this material exhibits an uptake of neutral hydrogen at high temperature and low oxygen partial pressure. The first theoretical analysis of hydration, electronic structure, and proton transport in A-doped Sr_2_Fe_1.5_Mo_0.5_O_6−δ_ (SFMO) perovskites was reported by Muñoz-García et al. [28]. They found that the K-doped SFMO is a promising oxygen ion-, proton-, and hole-conducting oxide material (so-called triple conducting oxide). The proton concentration in various perovskite-related structures containing iron or other transition metals were studied by several authors [29,30,31,32,33,34,35,36]. For example, Han et al. reported a significant proton concentration level of around 0.13 per unit cell in Sr and Fe co-doped LaScO_3_ [37], which is comparable with that reported for Y-doped barium zirconate [38]. A significant water uptake that was mostly related to the hydration process was observed in doped BaFeO_3_ materials. In 2017, Zohourian et al. [34] reported a proton concentration of around 0.11 per unit cell for Ba_0.85_La_0.15_Fe_0.8_Zn_0.2_O_3_ composition, which is actually one of the highest values reported for mixed conductors with high electron transfer numbers. Most of the reports show that the proton uptake occurs by OH^●^ incorporation (hydration), H_2_ incorporation (hydrogenation), or by a combination of the two processes [33,34].

In this work, SrTi_1−x_Fe_x_O_3−x/2−δ_ oxides with different content of Fe^3+^ (0 ≤ x ≤ 1) were synthesized and evaluated in dry and wet atmospheres. The main effort was devoted to the studies of structure, microstructure, water uptake, and proton concentration in these materials.

## 2. Materials and Methods 

### 2.1. Synthesis Protocol

The SrTi_1−x_Fe_x_O_3−x/2−δ_ samples with x = 0; 0.1; 0.2; 0.3; 0.4; 0.5; 0.6; 0.8; and 1 (in further text, these are denoted as STFx), were synthesized by a conventional two-step solid-state synthesis. Stoichiometric amounts of the SrCO_3_, TiO_2_, and Fe_2_O_3_ powders (all precursors with >99.9% purity) were mixed together in an agate mortar for 45 min. The mixed powders were uniaxially pressed into cuboidal pellets with an approximate size of 1 mm × 10 mm × 12 mm with a pressure of 200 MPa. In the first step of heat treatment, the pellets were heated at 1000 °C in the air for 12 h. In the next step, they were crushed, ground for 30 min, and repelletized using 300 MPa pressure, and sintered at a temperature between 1200 and 1400 °C for 24 h, depending on the composition. It has to be underlined that different sintering temperatures were checked for every sample in order to obtain single-phase materials. After that, the optimal temperature was chosen and listed in Table 1. The optimal sintering temperatures, as well as the measured densities and open porosities of the obtained pellets, are shown in Table 1.

### 2.2. Structural and Microstructural Characterization

In order to determine the structural properties of the obtained materials, a Phillips X’Pert Pro diffractometer (XRD) (Almelo, The Netherlands) with CuK_α_ (1.540 Å) was used. The measurements were carried out in the 10–120° range under 40 kV and 40 mA, at room temperature in air. Unit cell parameters were determined by Rietveld refinements in FullProf Suite software (3.0, June 2015) [39]. As an initial point of the analysis, unit cell parameters of the SrTiO_3_ structure (space group no. 221, *Pm-3m*) were used. 

The microstructure of the sintered pellets surfaces/cross-sections was examined on an FEI Quanta FEG 250 Scanning Electron Microscope (SEM) (Waltham, MA, USA). The SEM images were collected using an Everhart–Thornley Detector (ETD) detector for secondary electrons in a high vacuum at 20 kV acceleration voltage. The analysis of materials composition by Energy Dispersive X-Ray Spectroscopy (EDS) was performed using the EDAX ApolloX SDD spectrometer (Mahwah, NJ, USA). The density and porosity of the pellets were determined using the Archimedes method with kerosene as a liquid medium.

### 2.3. Thermogravimetric Analysis 

Thermogravimetric analysis (TGA) (Burlington, MA, USA) was performed to determine the oxygen nonstoichiometry and water uptake of SrTi_1−x_Fe_x_O_3−δ_. The analysis was conducted on a Netzsch Jupiter® 449 F1 both in dry and wet atmospheres. Two dry atmospheres were applied: dry air (pO_2_ ≈ 0.20 atm., pH_2_O ≈ 3 × 10^−5^ atm., estimated for RH = 80% and T = 25 °C in ProgasMix FC software (v 0.7.1)) and dry nitrogen (pO_2_ ≈ 2 × 10^−6^ atm., pH_2_O ≈ 3 × 10^−5^ atm.). In order to introduce water into the atmosphere, a gas mixer equipped with wetting and drying stages was used. Before each measurement, a blank run for baseline correction was carried out in both atmospheres separately. Prior to TGA tests, the samples were annealed in 1100 °C in the air for 10 h with a heating rate of 3 °C/min and quenched; for measurements, the pellets were ground.

To determine the oxygen vacancies concentration and oxidation enthalpy, the sample mass change was recorded in dry synthetic air and dry nitrogen in the temperature range of 40–1000 °C. Assuming that the recorded mass difference between the initial mass recorded at temperature *T*_0_ and the mass at temperature *T*, $\Delta m_{ox}(T)=m(T)-m(T_{0})$, is related to the oxygen loss only, the temperature evolution of the oxygen nonstoichiometry Δ*δ(T)_ns_* (molar oxygen vacancies concentration) as well as a volumetric concentration of oxygen vacancies related to the oxygen nonstoichiometry, ${\left[V_{O}^{••}\right]}_{ns}$, may be calculated according to the following relation:

(1) $$\Delta \delta {\left(T\right)}_{ns}=\delta \left(T\right)-\delta \left(T_{0}\right)=\frac{\mu_{s}\;\Delta m_{ox}\left(T\right)}{\mu_{o}m\left(T_{0}\right)}$$

where *µ_s_* and *µ_o_* are sample and atomic oxygen molar masses, respectively.

To determine the water uptake of SrTi_1−x_Fe_x_O_3−x/2−δ_, a series of TG analyses with switches between dry and wet air were performed. First, the sample was heated to 800 °C and held at this temperature for 180 min in dry air in order to remove adsorbed water and carbon dioxide from the surface. Next, the sample was cooled to 400 °C in dry air. After two hours, the dry air was switched into the humidified one (pH_2_O ≈ 2.3 × 10^−2^ atm.); then, after an additional 2 h, the wet air was switched back into the dry air. The difference between the masses recorded in the dry and wet air at 400 °C (Δ*m_H_*_2*O*_) allowed us to estimate the molar protonic defects concentration $\left[OH_{O}^{•}\right]$. Equation (5) gives the relations between Δ*m_H_*_2*O*_ and $\left[OH_{O}^{•}\right]$_hydrogen_ in the case of hydrogenation as a predominant process of forming protonic defects.

(2) $${\left[OH^{•}\right]}_{hydrogen}=\frac{2\;\Delta m_{H2O}\;\mu_{s}}{m_{s}\;\mu_{H2}}$$

where *µ_s_* is a molar mass of the sample and *µ*_*H2*_ is a molar mass of molecular hydrogen, whereas *m_s_* is the mass of the sample in dry air before the switch into the wet air.

## 3. Results

### 3.1. Structure and Microstructure of SrTi_1−x_Fe_x_O_3−x/2−δ_

**Figure 1:**
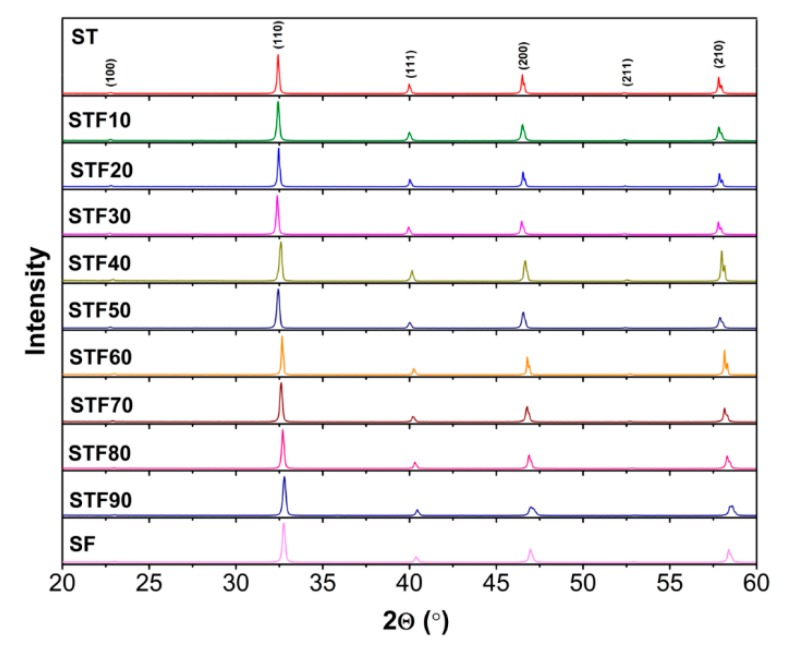
XRD patterns obtained for SrTi_1−x_Fe_x_O_3−x/2−δ_ solid solutions.

The X-ray diffractograms obtained for SrTi_1−x_Fe_x_O_3−x/2−δ_ samples are shown in Figure 1. All the observed diffraction reflections correspond to the cubic perovskite phase, which means that within the sensitivity of the XRD method, the samples in the wide iron content range are single-phase solid solutions of strontium titanate and ferrite. For Fe-rich compounds, this indicates that the substitution of Fe^4+^ by Ti^4+/3+^ cations in SrFeO_3_ stabilizes the perovskite cubic phase even for low Ti contents [40]. It was observed also in [25].

**Figure 2:**
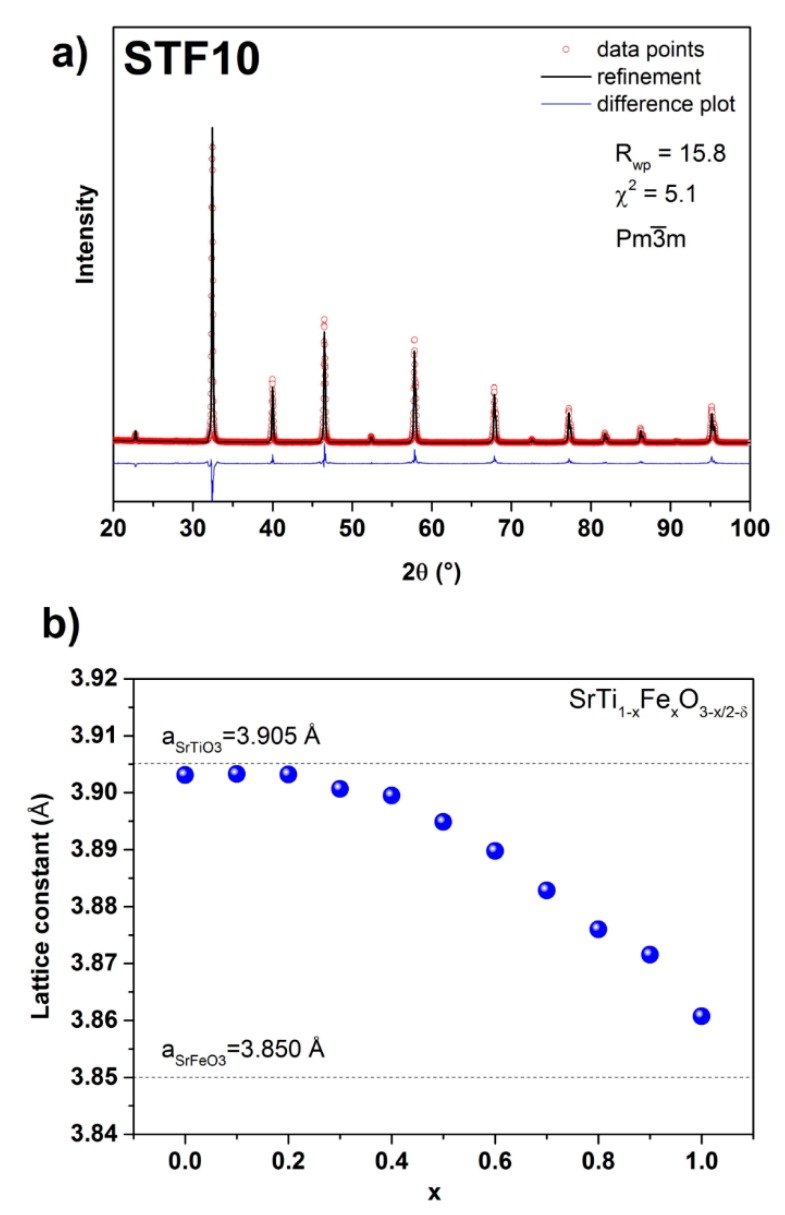
(**a**) Rietveld refinement profile for the SrTi_0.9_Fe_0.1_O_2.95−δ_ sample; (**b**) unit cell parameter as a function of iron content (x) in SrTi_1−x_Fe_x_O_3−x/2−δ_. Calculated values for SrTiO_3_ and SrFeO_3_ are shown as dashed horizontal lines.

The Rietveld refinements of the obtained RT diffractograms were carried out the model of a cubic perovskite structure with a Pm-3m space group. The exemplary results of the Rietveld profile obtained for SrTi_0.9_Fe_0.1_O_2.95−δ_ and the difference plot are shown in Figure 2a. The calculated values of unit cell parameters as a function of Fe content are presented in Figure 2b. The unit cell parameter remains constant until x ≈ 0.2, whereas for x > 0.2, it decreases with the increasing iron content. A similar tendency was observed by Vračar et al. [41] and Ghaffari et al. [42]. A quasi-linear dependence of the unit cell parameters on iron content, which follows Vegard’s rule, is caused by the difference between the ionic radius of six-fold coordinated Ti^4+^ cation (0.605 Å) and Fe^4+^ (0.585 Å) [43]. The substitution of Ti^4+^ with smaller Fe^4+^ affects the unit cell parameters indirectly, i.e. through its influence on the O^2−^ size, which occurs for x ≥ 0.15 [41]. This explains why Vegard’s rule is not observed in the samples with low iron content. Moreover, other factors also possibly influencing the oxygen sublattice such as grain size or different applied sintering temperatures may affect observed unit cell parameters. On the other hand, these factors, as introducing oxygen nonstoichiometry, are expected to cause the deviation from the linear dependence of unit cell parameter on x [41]. So, we considered the iron content in the SrTi_1−x_Fe_x_O_3−x/2−δ_ solid solutions as a major factor influencing the unit cell parameters. Another structural characteristic that may influence the properties of SrTi_1−x_Fe_x_O_3−x/2−δ_ is the possible lattice distortion caused by Ti substitution with Fe. Indeed, Vračar et al. [41] found that the EXAFS (Extended X-Ray Absorption Fine Structure) analysis showed the presence of different Ti-O and Fe-O distances in the same sample. This local lattice strain does not bring about a unit cell distortion: in the whole range of x, the Pm-3m space group appropriately describes the lattice. 

**Figure 3:**
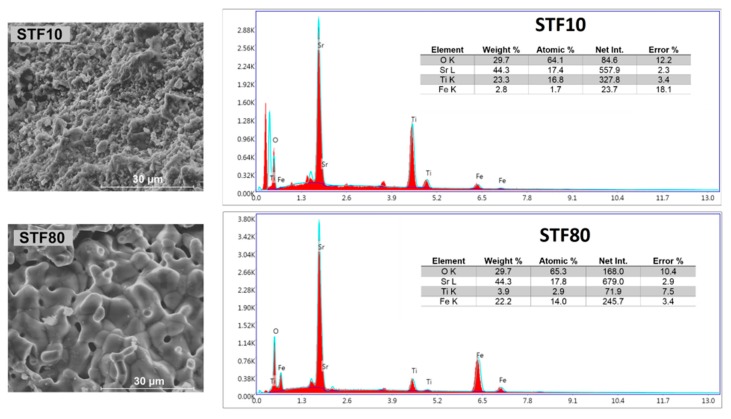
SEM micrographs and EDX spectra results of cross-sections of SrTi_1−x_Fe_x_O_3−x/2−δ_ for x = 0.1 and 0.8.

Figure 3 shows exemplary SEM images, as well as EDX spectra for SrTi_0.9_Fe_0.1_O_2.95−δ_ and SrTi_0.2_Fe_0.8_O_2.6−δ_ samples. All the investigated samples were porous, and the grain size differed as a function of iron content. The estimated grain sizes were between 0.5 and 14 µm, and the average value grew with increasing iron content. This may be caused by the faster diffusion of cations and oxygen during the synthesis in samples with larger Fe content. The Fe/Ti ratios, within an SEM-EDS measurement uncertainty of approximately 5%, are similar to the nominal values for two samples ((Fe/Ti)_nominal_ = 0.11; (Fe/Ti)_exp_ = 0.10 for STF10 and (Ti/Fe)_nominal_ = 0.25; (Ti/Fe)_exp_ = 0.21 for STF80).

### 3.2. Oxygen Nonstoichiometry

**Figure 4:**
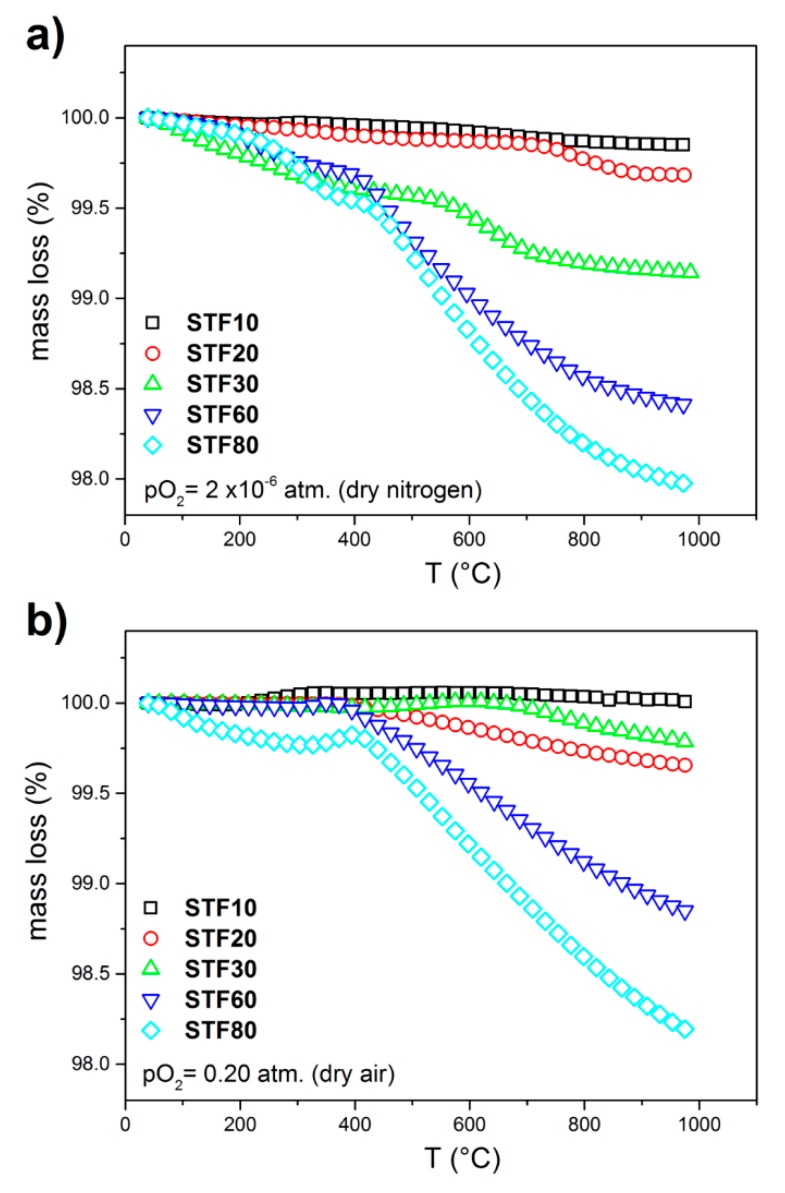
Mass loss in SrTi_1−x_Fe_x_O_3−x/2−δ_ as a function of temperature measured by thermogravimetric analysis (TGA) in dry nitrogen (**a**) and dry air (**b**). The heating rate for both atmospheres was 2 °C/min.

**Figure 5:**
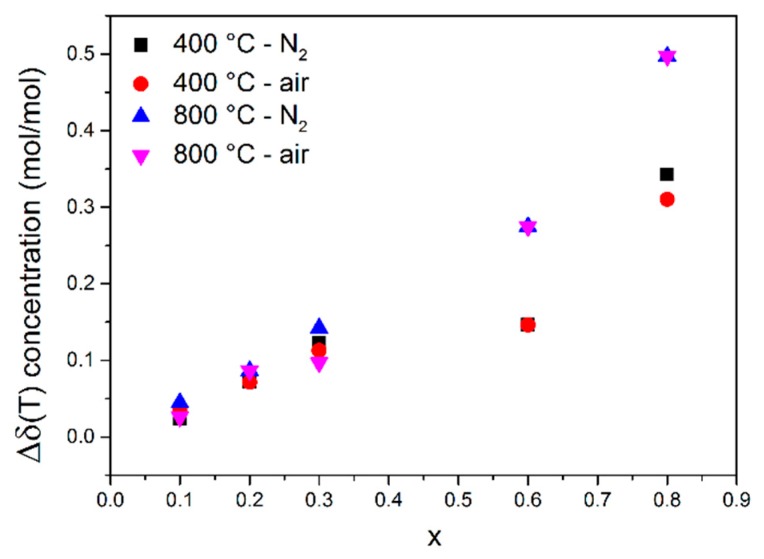
Total molar oxygen vacancy concentration $\Delta \delta {\left(T\right)}_{tot}$ as a function of iron content in SrTi_1−x_Fe_x_O_3−x/2−δ_ samples collected for two chosen temperatures: −400 and 800 °C.

The temperature dependence of mass loss as a function of temperature (200–1000 °C) in dry nitrogen and dry air atmospheres, collected by TG, is presented in Figure 4. Since the oxygen vacancies and electronic holes are predominant defects, the recorded mass change is related to the release of oxygen. The qualitative analysis of the plots obtained for particular samples reveals the following characteristic features: (1) The mass in dry nitrogen (Figure 5a) decreases monotonically with increasing temperature, whereas in the air (Figure 5b) in some temperature ranges, the mass is almost constant or even slightly increases. (2) In all cases, the rate of mass change depends on temperature. (3) The value of the total oxygen loss is higher in the samples with higher iron content. 

From the defect chemistry point of view, the heating of SrTi_1−x_Fe_x_O_3−x/2−δ_ in different atmospheres leads to the thermal generation of intrinsic defects, i.e., anion Frenkel defects, and equilibration of the oxygen content in the oxide with the partial oxygen pressure in the atmosphere. The former process is not observed in the TG results, since it does not change the oxygen stoichiometry, whereas the latter causes either oxygen deficiency (δ > 0) or excess (δ < 0), depending on the temperature and the oxygen pressure. As a result of the thermal history of the sample, a small “frozen-in” oxygen deficiency exists in the SrTi_1−x_Fe_x_O_3−x/2−δ_ oxides, which explains the small increase of oxygen content observed in the air at a temperature below approximately 500 °C. As could be expected, in the nitrogen atmosphere, only a depletion in oxygen occurs. The influence of the iron content on the rate of the mass change, the temperature onset of the mass change, as well as the maximum value of the mass change is related to the enthalpy of reduction and the chemical diffusion coefficient of oxygen in the SrTi_1−x_Fe_x_O_3−x/2−δ_ materials. It was reported that the reduction enthalpy is lower in the samples containing more iron [44], whereas the oxygen chemical diffusion coefficient is around two orders of magnitude higher in SrFeO_3_ than in SrTiO_3_ [45,46]. So, increasing the iron content causes an increase of both the rate of reduction reaction and the oxygen diffusion rate out of the oxide. Apart from the influence on the total oxygen nonstoichiometry, the diffusion constant strongly influences the temperature range at which a change in the oxygen release kinetics is different. Below some characteristic temperature (for example, 685 °C for STF20 or 550 °C for STF30 at dry nitrogen), probably only the oxygen release from the surface of the sample may be expected, whereas above this temperature, a combination of the releasing processes of oxygen may occur. A similar behavior was observed by Park et al. [47] for La-donor doped STF material and by Stevenson et al. in a (La,Sr)(Co,Fe)O_3_ system [48]. 

The data shown in Figure 4 allow estimating the total concentration of oxygen vacancies. As it was shown by e.g., Rothschild et al. [12] and Steinsvik et al. [14], in the whole range of iron content, iron prefers a +3 valence state in an oxidizing atmosphere; that is, the $Sr_{}^{2+}Ti_{1-x}^{4+}Fe_{x}^{3+}O_{3-x/2-\delta }$ formula is the most suitable to describe the STFx system. Therefore, Fe^3+^ is an acceptor dopant that is compensated predominantly by oxygen vacancies [12,25,49]. 

This can be expressed by Equation (3):

(3) $$Fe_{2}O_{3}\overset{SrTiO_{3}}{\to }2Fe_{Ti}^{\prime }+V_{O}^{••}+3O_{O}^{x}$$

A total molar oxygen vacancies concentration was calculated from Formula (4):

(4) $$\Delta \delta {\left(T\right)}_{tot}=\frac{1}{2}\left[Fe_{Ti}^{\prime }\right]+\Delta \delta {\left(T\right)}_{ns}$$

where $\Delta \delta {\left(T\right)}_{ns}$ is the molar oxygen vacancies concentration related to the oxygen nonstoichiometry, and $\left[Fe_{Ti}^{\prime }\right]$ denotes a molar acceptor dopant concentration. Figure 5 shows the obtained results as a function of iron content at two temperatures.

It can be seen that the increase of Fe content in the samples leads to an increase in a total molar oxygen vacancy concentration. In order to compare the obtained results to the values given in the literature, the volumetric total oxygen vacancies concentration was calculated. For this purpose, the suitable formula was used (Equation (5)):

(5) $${\left[V_{O}^{••}\right]}_{tot}=\frac{\Delta \delta {\left(T\right)}_{tot}}{V_{el}}$$

where *V_el_* is the volume occupied by one SrTi_1−x_Fe_x_O_3−x/2−δ_ formula unit, which in this case is the unit cell volume. The unit cell volume determined by XRD data at room temperature corrected on the basis of linear thermal expansion coefficient of pure and undoped SrTiO_3_ ($\alpha_{lin}=3.23\;\times \;10^{-5}K^{-1}$ [50]) was used for calculations in the case of all the investigated compositions. The isotropic conditions were assumed, meaning that the volumetric thermal expansion coefficient is three times larger than the linear one. The obtained values are in the range of 5.7 × 10^21^ to 1.1 × 10^23^ cm^−3^ depending on the temperature and composition and are in reasonably good agreement in comparison to the previously reported values by Rotschild et al. [12] (2.0 × 10^21^ cm^−3^ for STF50 at 900 °C).

### 3.3. Water Uptake and Proton Concentration Analysis

The formation of protonic defects in oxides may proceed through either hydration (Equation (6)) or hydrogenation (Equation (7)) processes. In the case of air atmosphere, these reactions can be expressed as:

(6) $$H_{2}O+V_{O}^{••}+O_{O}^{x}↔2OH_{O}^{•}$$

(7) $$H_{2}O_{\left(g\right)}+2O_{O}^{x}+2h^{•}↔1/2\;O_{2\left(g\right)}^{}+2OH^{•}$$

Generally speaking, the domination of a chemical reaction is mainly determined by a Gibbs free energy difference of reagents and products. This parameter is strongly correlated with a defect concentration. Thus, further analysis will be continued with respect to the defects concentration. The hydration requires the presence of the oxygen vacancies, whereas the hydrogenation requires the presence of holes. Equation (6) describes the hydration reaction, which means the incorporation of water by an acid–base reaction. This dominates when the concentration of oxygen vacancies is higher than that of electron holes 2$(\Delta \delta {\left(T\right)}_{ns})>p$. In the case of the reaction shown in Equation (7), it is basically a reaction in which water uptake is simultaneous with electronic carriers consumption. It dominates especially for materials with a relatively high concentration of electrons/holes −2$(\Delta \delta {\left(T\right)}_{ns})<p$. In the Fe^3+^-doped SrTiO_3_ system, an electroneutrality condition in the dry atmosphere can be described as:

(8) $$p+2{\left[V_{O}^{••}\right]}_{ns}=\left[Fe_{Ti}^{\prime }\right]+2\left[V_{Sr}^{\prime \prime }\right]$$

where *p* denotes the electron holes concentration, $\left[V_{Sr}^{\prime \prime }\right]$ denotes the volumetric strontium vacancy concentration, and ${\left[V_{O}^{••}\right]}_{ns}$ is the volumetric oxygen vacancy concentration related to the oxygen nonstoichiometry. In the temperature range studied in this work (25–1000 °C), the concentration of strontium vacancies may be assumed as negligible in comparison to *p* or $\left[V_{O}^{••}\right]$, because the formation energy of strontium vacancies (20.915 eV [51]) is much higher than that of redox enthalpy of oxygen (4.1–5.8 eV, depending on the iron content [12]). In the air, in acceptor-doped SrTiO_3_, the concentration of oxygen vacancies is much lower than that of electronic holes. For example, at 400 °C in air, in STF30, the molar oxygen vacancy concentration $\Delta \delta {\left(T\right)}_{ns}$ that is determined experimentally equals 0.002, while the hole molar concentration p calculated from the electroneutrality condition is as high as 0.296. The same calculations were performed for all the samples, and the obtained values showed that electronic holes are the majority defects. Thus, hydrogenation was assumed to be a predominant reaction. This is in line with findings by other researchers in similar systems [30,52,53,54].

**Figure 6:**
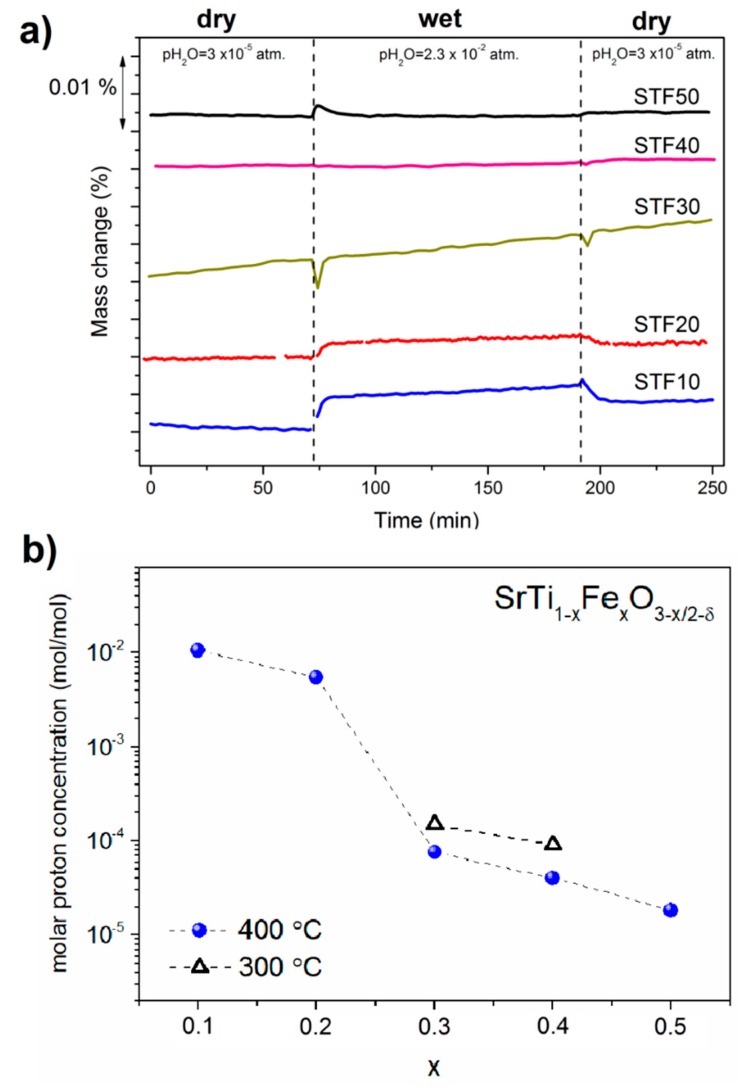
(**a**) Mass change recorded at the isothermal switch at 400 °C between dry (pH_2_O ≈ 3 × 10^−5^ atm.) and humidified air (pH_2_O = 0.023 atm.); (b) proton concentration at 400 °C and 300 °C versus iron content in SrTi_1−x_Fe_x_O_3−x/2−δ_.

Figure 6a presents water uptake measurements performed by TG. As can be seen, in all samples, before the switch from wet to dry, no significant mass change was observed, which indicates that the materials were equilibrated at a certain pO_2_ and temperature. As Figure 6 shows, introducing the water vapor into the atmosphere resulted in an increase in the mass of the samples. The mass change was observed immediately after the switch and then was followed by a slower process. After switching back the pH_2_O into the low values (dry conditions), again, a rapid mass loss was observed, but the final value in the dry gas was different from that at the initial state before the switch to wet. Such behavior suggests that in the wet atmosphere at the beginning, a much faster hydration process (Equation (6)) occurs, and then after a while, the much slower hydrogenation process (Equation (7)) takes place during the measurement. On the basis of the mass change during the switch from dry to wet conditions, a molar proton concentration was calculated for each sample under the assumption that hydrogenation is a predominant reaction of water uptake (see Equation (7)).

The proton concentration as a function of iron content presented for all compositions at 400 °C and for two chosen compositions (STF30 and STF40) at 300 °C is shown in Figure 6b. The obtained values at 400 °C are between 1.8 × 10^−5^ and 1.0 × 10^−2^ mol/mol and depend on the iron content. The values obtained at 300 °C are slightly higher—equal to 1.5 × 10^−4^ mol/mol for STF30 and 9.0 × 10^−5^ mol/mol for STF40, which is expected behavior in relation to the thermodynamic of protonation in oxides [55,56]. The highest concentration of protons measured at 400 °C—1.0 × 10^−2^ mol/mol—were obtained for the SrTi_0.9_Fe_0.1_O_2.95_ sample, whereas the lowest is noted for the SrTi_0.5_Fe_0.5_O_2.75_ composition and was equal to 1.8 × 10^−5^ mol/mol. For comparison, the value reported for Y-doped BaZrO_3_ is of the order of 10^−1^ at 400 °C, which is around one order of magnitude higher than in SrTi_0.9_Fe_0.1_O_2.95_. On the other hand, the obtained value for STF10 material at 400 °C is comparable with that reported for Ca-doped LaNbO_4_ [57].

Figure 6b shows that a molar proton concentration decreases with the increasing iron content in materials, whereas a characteristic and significant drop is observed above x = 0.2. As can be seen, the molar proton concentration in STF10 and STF20 is around two orders of magnitude lower than that in STF30. This is interesting, because when the iron content increases, the oxygen vacancies concentration also increases. In typical proton conductors, the increasing oxygen vacancy concentration promotes the water uptake and the proton concentration. Since the observations clearly show that in SrTiO_3−δ_-based materials the situation is opposite (Figure 6b), other factors than oxygen vacancy concentration should be taken into consideration. This also supports the hypothesis that hydrogenation, instead of typical acid–base hydration, is predominant in the analyzed materials.

**Figure 7:**
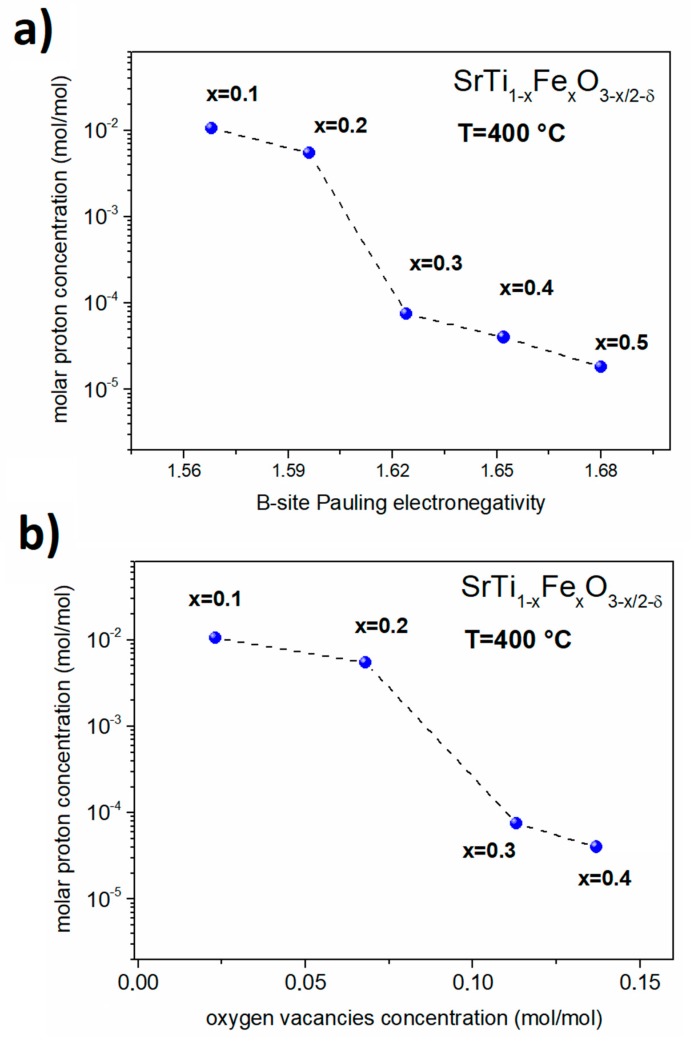
(**a**) Proton concentration at 400 °C plotted versus B-site Pauling electronegativity; (**b**) total oxygen vacancy concentration measured at 400 °C in air.

Iron in strontium titanate occupies B-sites, so that the analysis of the iron influence on the chemical nature of this site may help explain the observed properties. One of the factors that is known to be very important in the case of analysis of the hydration or hydrogenation of oxide is the basicity of cations [52,58]. Generally speaking, a higher basicity leads to more negative hydration/hydrogenation Gibbs energy ΔG_hydr_, which is favorable for proton uptake in oxides. Since the basicity is related to electronegativity, we analyzed the results using the Pauling electronegativity scale. B-site Pauling electronegativity $\chi_{B-site}$ was calculated for all compositions by the formula:

(9) $$\chi_{B-site}=(1-x)\cdot \chi_{Ti}+x\cdot \chi_{Fe}$$

where *x* denotes the iron content in materials; while $\chi_{Ti}=1.54$ and $\chi_{Fe}=1.83$ are electronegativities of titanium and iron, respectively [59]. The substitution of Fe in the Ti site decreases the basicity of the system because Fe is more electronegative in comparison to Ti. Therefore, as shown in Figure 7, molar proton concentration decreases with increasing B-site Pauling electronegativity—that is, with decreasing basicity. Increasing the electronegativity of (Ti_1−x_Fe_x_) may be also viewed in terms of the ionicity of the bonds between Ti(Fe) and O in BO_6_ octahedra. The lowering of proton uptake with increasing Fe content may be also related to the increased covalency of Ti(Fe)–O bonds. 

As it was mentioned, above the 20 mol% of iron substitution, a significant drop of proton molar concentrations was observed. Similar behavior can be seen in Figure 7a,b where the molar proton concentration was presented as a function of B-site electronegativity and oxygen vacancies concentration. As can be seen in Figure 4, the reduction of the samples with more than 20 mol% of iron is significant, and this may have an influence on the protonation. In mixed conducting oxides, the water uptake is related to the electronic structure and the amount of redox active cations. The protonation is strongly related to the concentration of delocalized electrons, as well as to the electron–oxygen vacancies interaction. These may lead to the lower hydration/hydrogenation [34,52,53]. In the case of the materials analyzed in this work, SrTi_1−x_Fe_x_O_3−x/2−δ_, the solid solution–electronic structure varies as a function of the Ti/Fe ratio. The valence band in STFx is mainly dominated by O 2p orbitals, whereas the conduction band is related to the Ti/Fe 3d orbitals. A redox state Fe^4+^/Fe^3+^ lies near the top of the valence band, and for higher Fe content, the width of this band increases, and the band starts to overlap of the electronic states of the O 2p states [60]. For low Fe concentrations, the hybridization of electronic iron orbitals and the other orbitals that are derived from Ti, O, and Sr is not strong, and then the orbitals do not significantly overlap [13]. This implies that there is no significant interaction between the electrons and oxygen vacancies, which would be detrimental for the water uptake, as was shown in [30]. That is why a relatively high proton concentration values for low Fe-content samples (STF10 and STF20) was noticed. Moreover, only a slight difference between the proton concentrations calculated for STF10 and STF20 can be seen. At an intermediate Fe-dopant concentration level (about 30–35%), which is very close to the percolation level in a doped STF system [61], the overlapping of the Fe and O orbitals may start to play an important role and may decrease the water uptake [30]. Moreover, in the case of approximately 35 mol% of Fe content, an interesting balance between the band-gap energy E_g_ and reduction enthalpy ΔH_red_ (directly related to the oxygen vacancies concentration) takes place and leads to the close to zero activation energy for a conduction mechanism (*E_a_* = *E_g_* − Δ*H_red_*/2) [13]. Below 35 mol%, the contribution from the concentration of charge carriers determines the temperature dependence of conductivity. When the concentration of iron exceeds approximately 35 mol%, the temperature dependence of the mobility of delocalized electrons from Fe cations is dominant [12,13]. Thus, this characteristic x = 0.3 point may explain a step decrease of the molar proton concentration between the STF20 and STF30 samples as well as a drop of a lattice parameter and a change of reduction kinetic for x > 0.2 (see Figure 2b and Figure 4b, respectively). At a higher concentration level (let’s say above 35 mol%), the overlapping of the electronic orbitals of oxygen and iron is significant, so the further increasing of the Fe content should lead to the low protons concentration and rather insignificant changes of this parameter. This also well agrees with the data shown in Figure 7.

As was shown in [12], the stronger overlap of Fe and O orbitals leading to valence band broadening as well as bandgap narrowing leads also to the weaker tendency to hydrogenation. According to this, we believe that the increasing electronegativity and orbitals overlapping of (Ti_1−x_Fe_x_) accompanying increasing x values is the main reason for the observed lower proton concentration in samples with higher Fe content.

## 4. Conclusions

The structure, microstructure, and water uptake were analyzed in a SrTi_1−x_Fe_x_O_3−x/2−δ_ cubic perovskite system synthesized by the conventional solid-state synthesis in a wide range of iron content (x = 0–1). The relation between the structure i.e., lattice parameter and composition, was analyzed, and it was found that this parameter changes nonlinearly with the Fe content in the sample. The microstructure of materials was checked by SEM. For the studied materials, different porosities and grain sizes (0.5–14 µm depending on the sample) were observed.

The oxygen nonstoichiometry, which is a crucial parameter needed to analyze and explain water incorporation in materials, was measured by TG at two atmospheres—dry air and dry nitrogen. The results showed that an oxygen vacancy concentration depends on the Fe amount, and this concentration increases for Fe-rich compositions. The determined values were in a range of 10^21^–10^23^ cm^−3^, which seems to be rational for such a group of acceptor-doped SrTiO_3_ materials. The water uptake of SrTi_1−x_Fe_x_O_3−x/2−δ_ was observed by using thermogravimetric studies. The collected mass changes allowed calculating the proton concentration. The values calculated per unit cell formula were found to be between 10^−5^ and 10^−2^ orders of magnitude, depending on the Fe/Ti ratio.

The relation between the materials’ stoichiometry (Fe/Ti ratio), oxygen vacancy concentration, and molar proton concentration in the studied samples were discussed. It was found that the increase of iron content leads to a significant decrease in molar proton concentration. It was also found and clearly indicates that not only an oxygen vacancy concentration but also factors such as the structure distortions, covalency of transition metal–oxygen bonds, as well as electron affinity of the chemical species present in materials play a major role in analyzing and explaining water uptake in Fe-doped SrTiO_3_ materials.

## Figures and Tables

**Table 1:** The SrTi_1−x_Fe_x_O_3−x/2−δ_ samples’ compositions, sintering temperature, densities, and porosities.

Sample	Sintering Temperature (°C)	Density (g cm^−3^)	Relative Density (%)
SrTiO_3_ (ST)	1400	4.57	89
SrTi_0.9_Fe_0.1_O_2.95_ (STF10)	1400	4.49	87
SrTi_0.8_Fe_0.2_O_2.9_ (STF20)	1400	4.81	92
SrTi_0.7_Fe_0.3_O_2.85_ (STF30)	1400	4.78	92
SrTi_0.6_Fe_0.4_O_2.8_ (STF40)	1300	3.53	67
SrTi_0.5_Fe_0.5_O_2.75_ (STF50)	1200	4.16	79
SrTi_0.4_Fe_0.6_O_2.7_ (STF60)	1200	4.45	84
SrTi_0.3_Fe_0.7_O_2.65_ (STF70)	1200	4.70	88
SrTi_0.2_Fe_0.8_O_2.6_ (STF80)	1200	4.12	76
SrTi_0.1_Fe_0.9_O_2.55_ (STF90)	1200	4.71	86
SrFeO_3_ (SF)	1200	4.70	85
